# A Pragmatic Device Based on a Double‐Sided Functional Structure for Efficient Water Harvesting

**DOI:** 10.1002/gch2.201900094

**Published:** 2020-01-31

**Authors:** Mingxue Chen, Zilin Yi, Shuang Tao, Shiyu Wang, Zhenggang Fang, Chunhua Lu, Zhongzi Xu

**Affiliations:** ^1^ State Key Laboratory of Materials‐Oriented Chemical Engineering College of Materials Science and Engineering Nanjing Tech University Nanjing 210009 P. R. China; ^2^ Jiangsu Collaborative Innovation Center for Advanced Inorganic Function Composites Nanjing Tech University Nanjing 210009 P. R. China; ^3^ Jiangsu National Synergetic Innovation Center for Advanced Materials (SICAM) Nanjing Tech University Nanjing 210009 P. R. China

**Keywords:** device, P(VDF‐HFP), radiative cooling, solar‐thermal technology, water collection

## Abstract

Water collection from fog has received much attention to meet the challenges of scarcity of clean drinking water in desert and arid regions. Currently, solar‐thermal technology is being used as an efficient, sustainable, and low‐cost method for water desalination to produce clean water. To collect the clean water, in recent years, most researchers have designed the structure of water collection surfaces. However, the heat released during the liquefaction process of droplets has an adverse effect on the condensation of droplets, and thus affecting the water collection efficiency. Here, in order to improve water collection efficiency, a radiative cooling layer is introduced on the back of the collection surface to dissipate the heat released during droplet liquefaction. The radiative cooling layer, consisting of poly(vinylidene fluoride‐co‐hexafluoropropene) embedded with SiO_2_ and CaMoO_4_ nanoparticles, can theoretically cool 18.1 °C below the ambient temperature in the daytime. With the addition of cooling coating on the back of the water collection surface, the water harvesting efficiency can be increased by 43–52%. The developed water harvesting device may provide a new pathway to the efficient collection of fresh water.

## Graphical abstract

A multifunctional, efficient water collection device based on a double‐sided functional structure is successfully fabricated. With the addition of radiative cooling coating (P(VDF‐HFP) embedded with SiO_2_ and CaMoO_4_ functional nanoparticles) on the back of the water collection surface, the water harvesting efficiency is increased by 43–52%.

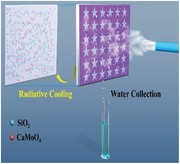

## 1 Introduction

With the growth of world economy and population, human demand for fresh water is increasing day by day. The shortage of freshwater is becoming a serious problem which has to be faced and solved.^1^, ^2^ Solar‐thermal technology, which involves the generation of vapor at temperatures lower than the boiling temperature and the generation of steam at or above the boiling temperature, has been widely used to generate clean water.^3^, ^4^, ^5^Recently, how to efficiently collect the clean water vapor on the surface of aluminum sheet to meet the challenge of the shortage of fresh water resources has become an important topic.^6^

In nature, some organisms have evolved different effective ways of getting water from the air. In recent years, some researchers have obtained effective water collection surfaces on aluminum by imitating the surface of organisms. For example, inspired by the lotus leaf for its large contact angle and small rolling angle to collect droplets easily,^7^ Zhang et al.^8^ prepared nano‐porous structure on pure aluminum surface by anodic oxidation. After stearic acid modification and drying and curing treatment, the contact angle of the aluminum surface can reach 154.6°. Liao et al.^9^ used CuCl_2_ solution and hydrochloric acid solution to etch aluminum in two successive steps to obtain micro–nano rough structure. After modification of cetyltrimethoxysilane, the superhydrophobic surface with a contact angle of 161.9° and rolling angle of 6.8° was obtained. It is energetically more conducive for water vapor and small water droplets to nucleate on hydrophilic surfaces compared to hydrophobic ones.^10^, ^11^, ^12^, ^13^ Inspired by the Namib desert beetle with the unique water harvesting ability, which uses patterned hydrophilic surface to nucleate and capture tiny water droplets in air for their survival, Yang et al.^14^ used NaCl solution to conduct a twice electrochemical‐etching method on aluminum, polyimide tape with cut different patterns used as mask, and the superhydrophilic patterns were finally prepared on the surface of fluoroalkysilane‐modified superhydrophobic aluminum sheet. The aluminum surface with superhydrophobic–superhydrophilic patterns showed excellent water harvesting efficiency. However, a problem which researchers have paid little attention is that some heat is emitted during the condensation of droplets. The heat released may reduce the liquefaction rate of droplets.^15^ Consequently, water collection efficiency is reduced.

Here, a cooling coating was added on the back of the highly efficient water collection aluminum surface modeled on a beetle. For droplet condensation that emits a lot of heat, adding a cooling coating on the back can keep the optimum temperature for droplet nucleation. The efficient radiative cooling coating is expected to have nearly unity infrared (IR) emissivity within 8–13 µm, which is known as the atmospheric window, in order to transfer the heat directly to outer space.^16^, ^17^, ^18^, ^19^ The significant radiative cooling at night by the cooling coating was achieved in previous work.^20^, ^21^, ^22^ In the daytime, since solar energy is absorbed strongly by the cooling coating, the enormous heat is generated on the surface of the coating.^23^, ^24^ Consequently, the efficient daytime cooling process requires cooling coating to have near‐uniform high solar reflectivity while emitting selectively and significantly within 8–13 µm region. Recently, Mandal et al.^25^ used phase‐inversion‐based method to make hierarchically porous polymers. Prepared P(VDF‐HFP) coating had a wide range of large and small pores (with a size range of ≈ 200 nm to 6 µm or larger), which together scatter all solar wavelengths efficiently. While the coating has excellent reflectance (R_solar_ ≥ 0.95), the coating exhibits selective emission peaks at atmospheric windows (8–13 µm) with the thickness less than 15 µm. In order to improve the cooling performance of the coating, we can enhance its emissivity in the atmospheric window.^24^ Due to the Si—O—Si asymmetric vibrations of SiO_2_ (8–10 µm)^26^ and the Mo—O stretching mode of CaMoO_4_ (11–13 µm),^27^ SiO_2_ and CaMoO_4_ functional nanoparticles were embedded in the poly(vinylidene fluoride‐co‐hexafluoropropene) P(VDF‐HFP) film to match well with an atmosphere window. Because of the addition of the P(VDF‐HFP) composite coating, the water collection efficiency can be increased by 43–52%. This strategy reported here can be widely adopted for the efficient water collection, which takes a key step on the practical applications in the water harvesting field.

## 2 Results and Discussion

**Figure 1:**
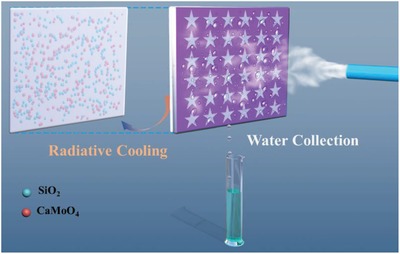
Schematic of a water harvesting device with radiative cooling coating on the back of the water collection surface.

In this work, a multifunctional efficient water collection device had been successfully fabricated (**Figure** **1**). One side of the Al sheet is an efficient water harvesting surface, which spread all over the star‐shaped wettability patterns. The other side of the Al sheet is a P(VDF‐HFP) cooling coating embedded with SiO_2_ and CaMoO_4_ nanoparticles. The addition of the P(VDF‐HFP) composite coating on the back of the water collection surface is aimed to improve water harvesting efficiency.

### 2.1 Water Collection Surface

**Figure 2:**
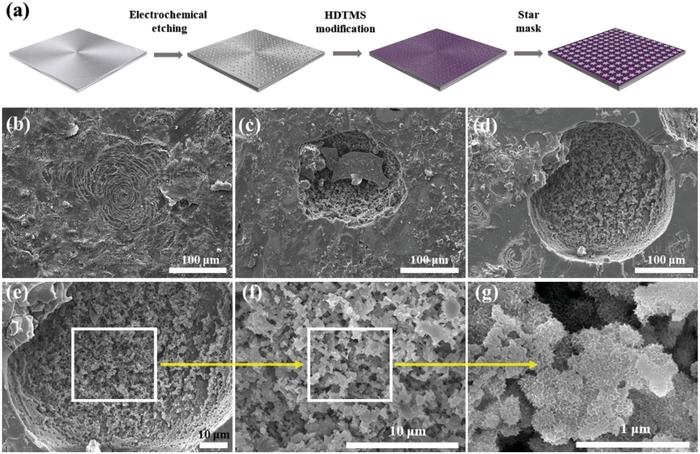
a) Schematic illustration for the fabrication process of the star‐shaped wettability patterns. SEM images of the Al sheet that was electrochemically etched at 0.16 A cm^−2^ for b) 1 min, c) 5 min, d–g) 11 min.

The star‐shaped wettability patterns were fabricated on the basis of a facile procedure as shown in **Figure** **2**a, which consists of electrochemical etching, hexadecyl trimethoxysilane (HDTMS) modification, mask attachment, and twice electrochemical etching. Figure 2b–g shows the SEM images of Al surface etched at 0.16 A cm^2^ for different time. After the electrochemical etching, a series of randomly distributed micron‐sized holes and stepped structure were produced on the surface of aluminum. When an electrochemical current was applied between the surface and the cathode, Al surface was gradually etched. Due to the short etching time, the surface was not completely etched, forming the stripes of holes (Figure 2b), which was etched only 1 min. With the etching time increasing to 5 min, the holes were practically formed. However, some un‐etched area in the holes can be distinguished in the SEM images (Figure 2c). After etching for 11 min, the micron holes were formed on the surface of Al sheet (Figure 2d–g). Because Al has good crystallinity and contains many trace elements, the reaction points on its surface have different velocities, and then anisotropic etching occurs, and a series of heterogeneous holes are formed (Figure 2c). As shown in Figure 2d,e, the boundary between the substrate and holes is clearly demarcated. The needle‐like nano structures are distributed uniformly on step‐like micro structures (Figure 2f,g), which can wrap up the air and provide the general condition for the formation of superhydrophobicity.

**Figure 3:**
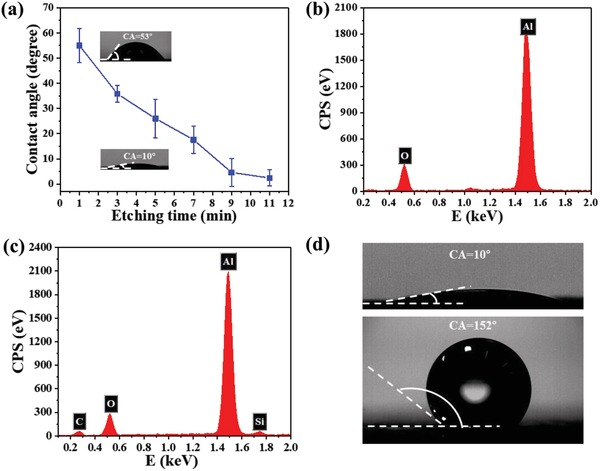
a) Variation of surface wettability as a function of electrochemical processing time etched at 0.16 A cm^−2^. b) EDS spectrum of the etched surface. c) EDS spectrum of the surface modified by cetyltrimethoxysilane. d) Contact angle of the surfaces of the star‐shaped patterns (up) and the rest area modified by HDTMS (down).

It can be seen from **Figure** **3**a that the contact angle of the etched surface decreased with the increase of etching time. Al surface became superhydrophilic after electrochemical etching for 11 min. Figure 3b shows the EDS spectrum of the etched surface, and element Al, O can be detected. The hydrophilic Al_2_O_3_ was formed on the surface of the aluminum sheet after etching. With the increment of etching time, the surface roughness increased, as shown in Figure 2b–g. According to Young's equation, the contact angle of the original hydrophilic material decreases with the increase of roughness.

After modification with cetyltrimethoxysilane, the EDS spectrum of the superhydrophobic Al surface reveals the presence of C, O, Al, and Si (Figure 3c). Silane molecule has been considered to be attached to the metal substrate by the hydrolysis of silane species with surface functional groups (Al‐OH), which forms the self‐assembled polysiloxane monolayer with low surface energy in the end.^28^ The appearance of C and Si indicates that the Al surface has been covered with silane film, and the C long‐chain has been successfully inserted on the surface.

Finally, a star‐shaped stainless steel mask was used to conduct a twice electrochemical etching. Superhydrophobic surfaces with superhydrophilic patterns were obtained after taking away the mask. The CA of star‐shaped patterns became 10° (Figure 3d) when the mask attached surface was etched again. And the rest of the surface maintained the original superhydrophobicity; the CA was 152° (Figure 3d).

### 2.2 P(VDF‐HFP) Composite Coating

**Figure 4:**
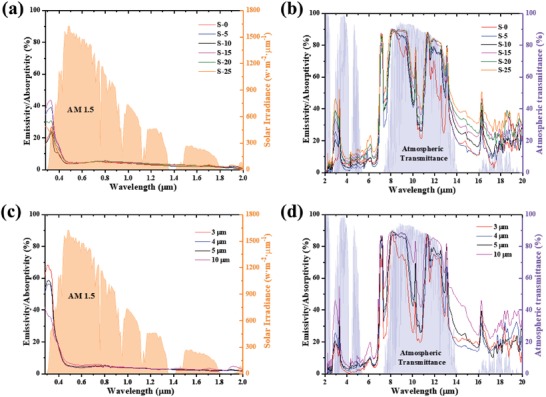
a) Solar absorptivity and b) IR emissivity/absorptivity of the samples with a different nanoparticle mass fraction. c) Solar absorptivity and d) IR emissivity/absorptivity of the samples with different film thicknesses.

As shown in **Table** **1**, the P(VDF‐HFP) composite coatings with different nanoparticle mass fraction were prepared to optimize the infrared emission characteristics. T_a_ and T_r_ in Table 1 are the ambient and the emitter temperature, respectively. **Figure** **4**a illustrates the solar absorptivity of these samples, which is necessary for the evaluation of the cooling performance of these samples. Due to the high reflectivity of aluminum sheets and the limited solar absorptivity of the P(VDF‐HFP) film, the solar absorptivity of all samples is limited, which is essential for effective daytime cooling. Figure 4b shows that the IR emissions within and outside the atmospheric window are both enhanced with the increase of the nanoparticle mass fraction. The solar irradiance I_AM_1.5(λ) is ASTM G173‐03 Reference Spectra derived from SMARTS v. 2.9.2 (AM1.5) and the atmospheric transmittance t(λ) is calculated using MODTRAN 5 with a relative humidity of 60%.^29^

**Table 1:** Samples with different nanoparticle mass fractions and their corresponding theoretical cooling performance

Samples	S‐0	S‐5	S‐10	S‐15	S‐20	S‐25
Mass fraction of SiO_2_ [%]	0	5	10	15	20	25
Mass fraction of CaMoO_4_ [%]	0	5	10	15	20	25
Ta‐Tr [°C]	12.3	13.9	18.1	17.6	15.2	14.3

The sample S‐10 was selected to prepare films with different thicknesses due to its best theoretical cooling efficiency. As shown in Figure 4c, all the samples have limited solar absorptivity. Figure 4d shows that when the thickness of the film increases, the IR emissions within and outside the atmospheric window are both enhanced.

**Figure 5:**
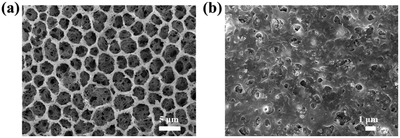
SEM images of a) P(VDF‐HFP) coating and b) P(VDF‐HFP) coating embedded with SiO_2_, CaMoO_4_ functional nanoparticles.

**Figure** **5** is the SEM images of P(VDF‐HFP) coating before and after being embedded functional particles. Pure P(VDF‐HFP) coating is hierarchically porous. The size of the big pores is around 5 µm and the small pores is about 200 nm (Figure 5a). From an optical point of view, the large and small holes together effectively scatter all the wavelength of sunlight. The SiO_2_ and CaMoO_4_ functional nanoparticles were filled in the pores of P(VDF‐HFP) coating (Figure 5b).

In order to determine the optimal mass fraction of functional nanoparticles and film thickness, the theoretical cooling performance was evaluated. The net cooling power of a radiative cooler (P_net_) could be calculated as follows:

(1) $${\text{P}}_{\text{net}}={\text{P}}_{\text{r}}-{\text{P}}_{\text{a}}-{\text{P}}_{\text{nonrad}}-{\text{P}}_{\text{sun}}$$

where

(2) $${\text{P}}_{\text{r}}=2\pi \int_{0}^{\pi \text{/}2}\text{sin}\theta \;\text{cos}\theta \;\text{d}\theta \;\int_{0}^{\infty }\text{B}\left({\text{T}}_{\text{r}},\lambda \right){\text{e}}_{\text{r}}\left(\lambda ,\theta \right)\text{d}\lambda$$

is the radiative output power of the coating.

(3) $${\text{P}}_{\text{a}}=2\pi \int_{0}^{\pi \text{/}2}\text{sin}\theta \;\text{cos}\theta \;\text{d}\theta \;\int_{0}^{\infty }\text{B}\left({\text{T}}_{\text{a}},\lambda \right){\text{e}}_{\text{a}}\left(\lambda ,\theta \right){\text{e}}_{\text{r}}\left(\lambda ,\theta \right)\text{d}\lambda$$

is the amount of the incident radiation power absorbed by the coating.

In the above formula, B(T, λ) = $\frac{2{\text{hC}}^{2}}{\lambda^{5}({\text{e}}^{\frac{\text{hC}}{\lambda \text{KT}}}-1)}$ is the spectral radiance of a black body at any temperature T depending on Planck's law, where C represents the speed of light in vacuum, h represents the Plank constant, K represents the Boltzmann constant, and λ represents the wavelength. According to Kirchhoff's law, the emissivity of the cooling coating is equal to its absorptivity e_r_(λ,θ). e_a_(λ,θ), which was defined by e_a_ (λ,θ) = 1 − t(λ)^1/cosθ^,^23^ is the angle dependent emissivity of the atmosphere.

In Equation (1), P_nonrad_ is nonradiative heating power got by the coating from the surrounding media, which can be defined as:

(4) $${\text{P}}_{\text{nonrad}}=\text{q}\left({\text{T}}_{\text{a}}-{\text{T}}_{\text{r}}\right)$$

here, q = q_conduct_ + q_convection_ is combined nonradiative heat coefficient taking root in the conductive and convective heat exchange of the coating with the surrounding air. From previous studies, we can see that q varies from 0 to 6.9 W m^−2^ K^−1^ with the change of environment and testing equipment.^19^, ^30^

The last one on the right side of Equation (1) is the solar power absorbed by the coating, which can be defined as

(5) $${\text{P}}_{\text{sun}}=\int_{0}^{\infty }{\text{e}}_{\text{r}}\left(\lambda ,\theta_{\text{sun}}\right){\text{I}}_{\text{AM}\;1.5}\left(\lambda \right)\text{d}\lambda$$

e_r_(λ,θ_sun_) is the absorptivity of the coating which has a bearing on the solar incident angle θ_sun_.

The daytime cooling power of a cooling coating is illustrated in Equation (1). If a positive value of P_net_ is achieved in the initial state (T_a_ = T_r_), the coating can be defined by a daytime cooling film. Moreover, the temperature difference T_a_ − T_r_ is expected to reach a steady state when the outgoing power keeps balance with the absorbed incoming power (P_net_ = 0), which means there is no extra power for the coating to further cool down. Consequently, the value of T_a_ − T_r_ in a steady state can be used to carry out the quantitative study on the cooling performance of the coating.

**Figure 6:**
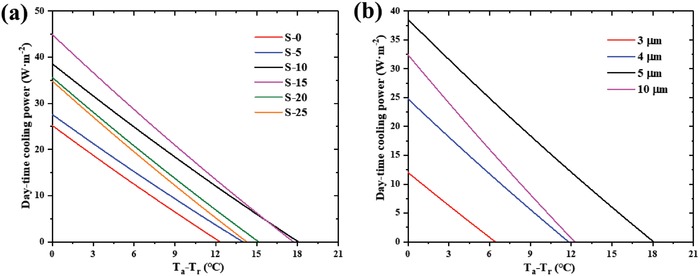
Theoretical daytime net cooling power of a) the samples with a different nanoparticle mass fraction, b) the samples with different film thicknesses.

The steady state temperature difference T_a_ − T_r_ of the composite coatings with different nanoparticle mass fractions under direct sunlight (AM 1.5) is shown in Table 1. The ambient temperature was set to 300.15 K and both the conductive and convective heat transfer were eliminated (q = 0 W m^−2^ K^−1^). It can be seen that T_a_ − T_r_ increases with the increasing nanoparticle mass fraction when the mass fraction is relatively low. However, T_a_ − T_r_ decreases with the increasing nanoparticle mass fraction when the mass fraction is relatively high. Table 1 shows that the sample S‐10 exhibited the best cooling performance. **Figure** **6**a shows the theoretical daytime cooling performance of the samples with different nanoparticle mass fraction. Then, the sample S‐10 was selected for further research. **Table** **2** shows T_a_ − T_r_ of the samples with different film thicknesses. As can be seen, for the samples that contain 10% (mass fraction) SiO_2_ and 10% (mass fraction) CaMoO_4_, the optimal thickness is 5 µm. Figure 6b shows the theoretical daytime cooling performance of the sample S‐10 with different film thicknesses. In order to test the actual cooling effect of the coating, S‐10 was selected for further investigation.

**Table 2:** Theoretical daytime cooling performance of the samples with different film thicknesses

Sample Thickness [µm]	3	4	5	10
T_a_‐T_r_ [°C]	6.4	11.8	18.1	12.3

### 2.3 Water Harvesting Performance

**Figure 7:**
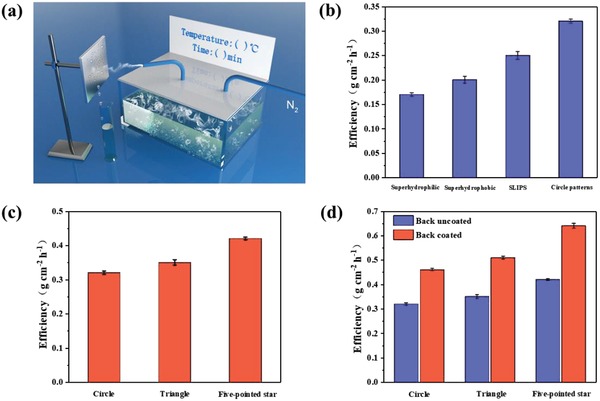
a) Schematic illustration of the setup used to measure the water collection efficiency of different samples. Water harvesting efficiency of different surfaces: b) the superhydrophilic surface, superhydrophobic surface, SLIPS surface, and circular‐patterns surface; c) the circular‐pattern surface, triangle‐pattern surface, and five‐pointed star surface; d) the circular‐pattern surface, triangle‐pattern surface, and five‐pointed star surface coated with/without cooling coating, respectively.

**Figure** **7**a is a schematic diagram of the water collection device. Aluminum sheet was fixed on the iron rack, the constant‐temperature water bath container was heated to 90 °C to form water vapor, and then the water bath container is sealed with two air holes. To generate a steady steam, the nitrogen flow was maintained with a constant velocity ≈20 L min^−1^. The condensed water droplets on the aluminum sheet fell with gravity, and the whole process was recorded by means of electronic scales.

It is considered that fog collection efficiency is determined by three main steps: capture, collection, and transmission. In the first step, fog capturing is a water collection process where fog droplets liquefy on the water collection surface and emit heat. In order to solve the effect of heat on droplet condensation, a cooling coating was added to the back of the surface to eliminate the adverse effect of temperature rise on droplet liquefaction. In our measurements, we used an infrared camera to measure the surface, fog, and atmosphere temperatures. During the collection and transmission steps, it is more favorable for water vapor and small water droplets to nucleate on the hydrophilic surfaces compared to hydrophobic ones. For transmission, transport capacity of hydrophobic surface is greater than that of the hydrophilic surface. As a result, an ideal surface has to own efficient droplet nucleation and water removal.

Based on the above discussions, firstly we investigated the water collection ability of circular‐shaped wettability patterns comparing to bare superhydrophilic surface, bare superhydrophobic surface, and SLIPS surface (Figure 7b). The uniformly superhydrophobic surface collects more water than the uniformly superhydrophilic surface (≈0.17 and ≈0.20 g cm^−2^ h^−1^), because the droplets roll more easily on the superhydrophobic surface instead of adhering and evaporating on the superhydrophilic surface. The surface, known as slippery liquid‐infused porous surface (SLIPS), was made to study its water collection ability. In agreement with the results in the literature,^31^ the SLIPS surface shows better water collection ability (≈0.25 g cm^−2^ h^−1^) than either uniformly superhydrophilic or superhydrophilic surface for its excellent droplet removal capabilities. Not unexpectedly, it was demonstrated that the water harvesting efficiency of the resultant stenocara beetle‐inspired circle‐patterned surfaces (≈0.32 g cm^−2^ h^−1^) was superior to that of the SLIPS (Figure 7b).

To study on water collection performance of different wetting patterns, secondly we investigated the water collection ability of circle, triangle, and five‐pointed stars. It was demonstrated that the water harvesting efficiency is a parabolic relationship with superhydrophilic density on the surface.^13^ Hence, the superhydrophilic areas on these designed surfaces are pretty close (20.97%, 20.02%, 20.50%, respectively). As shown in Figure 7c, the surface with five‐pointed star patterns collected more water (≈0.42 g cm^−2^ h^−1^) than that with triangle patterns (≈0.35 g cm^−2^ h^−1^). It is remarkable that both star‐shaped and triangle‐shaped patterns were more efficient than circular‐shaped patterns (≈0.32 g cm^−2^ h^−1^). This can be attributed to the integration of both the wettability gradient^32^, ^33^, ^34^ and shape gradient^35^, ^36^, ^37^ on the surface, which together accelerate the process by directionally collecting water droplet toward more wettable regions.

**Figure 8:**
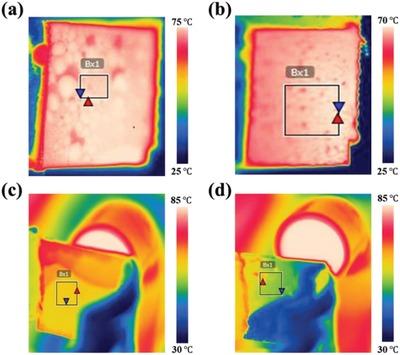
Infrared thermal images of different surfaces. a) Water collection surface uncoated P(VDF‐HFP) composite coating. b) Water collection surface coated P(VDF‐HFP) composite coating. c) The back of the water collection surface coated P(VDF‐HFP) composite coating. d) The back of the water collection surface uncoated P(VDF‐HFP) composite coating.

In order to reduce the influence of heat release during droplet liquefaction on steam condensation, finally the P(VDF‐HFP) coating embedded with 10% SiO_2_ (mass fraction) and 10% CaMoO_4_ (mass fraction) was introduced on the back of the water collection surface. When the constant‐temperature water bath was heated to 90 °C, the temperature of the water vapor to the water collection surface was about 80 °C. As can be seen in **Figure** **8**a, the temperature of the water collection aluminum surface was about 75 °C. However, if the radiative cooling layer S‐10 was attached on the back of water collection surface, the temperature of water collection surface was about 71 °C (Figure 8b). Hence, the temperature reduction was about 4 °C. The P(VDF‐HFP) composite coating absorbed the condensation heat and emitted it. As a result, the temperature of the back of water collection surface which was coated P(VDF‐HFP) composite coating was about 64 °C (Figure 8c), which was higher than that of the uncoated one (Figure 8d). As shown in Figure 7d, the water collection efficiency of the circle, triangle, and five‐pointed star patterned surfaces with cooling coatings show 43.75%, 45.71%, and 52.38% improvement, respectively, compared to the uncoated ones.

## 3 Conclusion

In summary, inspired by cooperative hydrophilic/hydrophobic regions of the Namib Desert beetle, the superhydrophilic/superhydrophobic patterned surfaces were fabricated on aluminum sheet using a twice electrochemical‐etching method for efficient water harvesting. Fog collection efficiency of the superwettable‐patterned samples was better than bare superhydrophilic surface, bare superhydrophobic surface, and SLIPS surface. Five‐pointed star patterned surface showed the best water collection ability for the integration of both wettability gradient and shape gradient on the surface, which together accelerate the process by directing tiny droplets to more wettable areas. Furthermore, P(VDF‐HFP) coating embedded with SiO_2_ and CaMoO_4_ functional nanoparticles on the back of the water collection surface to release the heat emitted by small droplets during atomization. As a result, water collection efficiency showed 43–52% increment than that of the original uncoated surface. These results demonstrate that the device with the introduction of radiative cooling coating on the back of the water collection surface provides a more efficient way to the collection of fresh water.

## 4 Experimental Section

##### Materials

All chemicals were used without further purification in the experiments. Aluminum sheets (2.5 × 2.5 × 0.4 cm^3^ in size) were purchased from Shenzhen Dixuan Metal Co., Ltd (China). NaCl (purity ≥99.5%) were purchased from Xilong Science Co., Ltd (China). Cetyltrimethoxysilane (CH_3_(CH_2_)_15_Si(OCH_3_)_3_; purity ≥95%) was obtained from Shanghai Bide Pharmatech Ltd (China). Silicone oil was obtained from Zhejiang Yiwu Keyuan Chemical Co., Ltd (China). P(VDF‐HFP) was supplied from Sigma‐Aldrich Shanghai Trading Co., Ltd (China).

##### Preparation of the Water Collection Surface with Star‐Shaped Wettability Patterns

The pure aluminum sheet was electrochemically etched (anode, aluminum; cathode, platinum tablet) in a neutral electrolyte of 0.2 mol L^−1^ NaCl with the electric current density of 0.16 A cm^−2^ for 11 min, and the etched surface then became superhydrophilic with dense micron‐steps and nano‐sized holes. The etched Al sheet was immersed in 2% HDTMS ethanol solution for 1 h to reduce the surface energy, then dried at 80 °C for 30 min. After that, the surface was converted into superhydrophobic. A stainless steel sheet with star‐shaped patterns as a mask was firmly attached to the superhydrophobic surface. The masked surface was etched again at 0.16 A cm^−2^ for 11 min in a 0.2 mol L^−1^ NaCl solution. Taking off the stainless steel sheet, superhydrophobic surfaces with superhydrophilic patterns were obtained.

##### Preparation of P(VDF‐HFP) Composite Coating

For producing the functional thin film, 2 g P(VDF‐HFP) raw material was first dissolved in 35 g acetone. Then, the SiO_2_ and the CaMoO_4_ nanoparticles were added to the solution. After ball‐milling for 24 h, the nanoparticles were randomly dispersed into the P(VDF‐HFP) solution. Lastly, the composite coating was prepared on the flat aluminum sheet by blade coating.

##### Characterization

An optical contact angle (CA) meter (JGW‐360A, China) was used to measure the CA of the surface. A scanning electron microscope (SEM, Hitachi SU8010, Japan) and an energy dispersive spectrometer (EDS, Hitachi SU8010, Japan) were used to study the micro structures of the etched surface and the chemical composition of the prepared samples, respectively. The thickness of the functional thin film of these samples was measured by the coating thickness gauge (Fisher MPO, Bad Salzuflen, Germany). The solar and IR reflectivity R(λ) were measured using the UV–Vis–NIR Spectrometer (SHIMADZU, Kyoto, Japan) and Fourier transform infrared spectrometer (FT‐IR, Frontier, PerkinElmer LLC), respectively. The thermal images were taken by an infrared thermal imaging camera (FLIR A615).

## Conflict of Interest

The authors declare no conflict of interest.

## References

[1] J. T. Overpeck , Nature 2013, 503, 350. 24256801 10.1038/503350a

[2] D. S. Liu , Nature 2016, 537, 307. 10.1038/537307c 27629629

[3] M. L. Roderick , G. D. Farquhar , Science 2002, 298, 1410. 12434057 10.1126/science.1075390

[4] A. Ohmura , M. Wild , Science 2002, 298, 1345. 12434040 10.1126/science.1078972

[5] H. M. Qiblawey , F. Banat , Desalination 2008, 220, 633.

[6] H. Kim , S. Yang , S. R. Rao , S. Narayanan , E. A. Kapustin , H. Furukawa , A. S. Umans , O. M. Yaghi , E. N. Wang , Science 2017, 356, 430. 28408720 10.1126/science.aam8743

[7] L. Feng , Y. A. Zhang , J. M. Xi , Y. Zhu , N. Wang , F. Xia , L. Jiang , Langmuir 2008, 24, 4114. 18312016 10.1021/la703821h

[8] Y. F. Zhang , X. Q. Yu , H. Wu , J. Wu , Appl. Surf. Sci. 2012, 258, 8253.

[9] R. J. Liao , Z. P. Zuo , C. Guo , Y. Yuan , A. Y. Zhuang , Appl. Surf. Sci. 2014, 317, 701.

[10] A. R. Parker , C. R. Lawrence , Nature 2001, 414, 33. 11689930 10.1038/35102108

[11] X. L. Liu , P. Cheng , Int. J. Heat Mass Transf. 2015, 83, 83842.

[12] Y. M. Hou , M. Yu , X. M. Chen , Z. K. Wang , S. H. Yao , ACS Nano 2015, 9, 71. 25482594 10.1021/nn505716b

[13] H. Bai , L. Wang , J. Ju , R. Z. Sun , Y. M. Zheng , L. Jiang , Adv. Mater. 2014, 26, 5025. 24847736 10.1002/adma.201400262

[14] X. L. Yang , J. L. Song , J. K. Liu , X. Liu , Z. J. Jin , Sci. Rep. 2017, 7, 12.28144037

[15] J. Ju , H. Bai , Y. M. Zheng , T. Y. Zhao , R. C. Fang , L. Jiang , Nat. Commun. 2012, 3, 6. 10.1038/ncomms2253 PMC3535335 23212376

[16] T. S. Safi , J. N. Munday , Opt. Express 2015, 23, A1120. 26406742 10.1364/OE.23.0A1120

[17] C. An , J. Su , Appl. Therm. Eng. 2011, 31, 2508.

[18] M. M. Hossain , B. H. Jia , M. Gu , Adv. Opt. Mater. 2015, 3, 1047.

[19] C. J. Zou , G. H. Ren , M. M. Hossain , S. Nirantar , W. Withayachumnankul , T. Ahmed , M. Bhaskaran , S. Sriram , M. Gu , C. Fumeaux , Adv. Opt. Mater. 2017, 5, 1700460.

[20] P. G. Scowcroft , F. C. Meinzer , G. Goldstein , P. J. Melcher , J. Jeffrey , Restor. Ecol. 2000, 8, 161.

[21] A. R. Gentle , G. B. Smith , Nano Lett. 2010, 10, 373. 20055479 10.1021/nl903271d

[22] A. R. Gentle , J. L. C. Aguilar , G. B. Smith , Sol. Energy Mater. Sol. Cells 2011, 95, 3207.

[23] A. P. Raman , M. A. Anoma , L. Zhu , E. Rephaeli , S. Fan , Nature 2014, 515, 540. 25428501 10.1038/nature13883

[24] M. M. Hossain , M. Gu , Adv. Sci. 2016, 3, 1500360. 10.1002/advs.201500360 PMC5067572 27812478

[25] J. Mandal , Y. K. Fu , A. C. Overvig , M. X. Jia , K. R. Sun , N. N. Shi , H. Zhou , X. H. Xiao , N. F. Yu , Y. Yang , Science 2018, 362, 315. 30262632 10.1126/science.aat9513

[26] A. Sakthisabarimoorthi , S. Dhas , M. Jose , Mater. Sci. Semicond. Process. 2017, 71, 69.

[27] S. Vidya , S. Solomon , J. K. Thomas , Phys. Status Solidi A 2012, 209, 1067.

[28] Y. Liu , X. M. Yin , J. J. Zhang , Y. M. Wang , Z. W. Han , L. Q. Ren , Appl. Surf. Sci. 2013, 280, 845.

[29] L. X. Zhu , A. P. Raman , S. H. Fan , Proc. Natl. Acad. Sci. USA 2015, 112, 12282. 26392542 10.1073/pnas.1509453112 PMC4603484

[30] H. Bao , C. Yan , B. X. Wang , X. Fang , C. Y. Zhao , X. L. Ruan , Sol. Energy Mater. Sol. Cells 2017, 168, 78.

[31] T. S. Wong , S. H. Kang , S. K. Y. Tang , E. J. Smythe , B. D. Hatton , A. Grinthal , J. Aizenberg , Nature 2011, 477, 443. 21938066 10.1038/nature10447

[32] M. K. Chaudhury , G. M. Whitesides , Science 1992, 256, 1539. 17836321 10.1126/science.256.5063.1539

[33] S. Daniel , M. K. Chaudhury , J. C. Chen , Science 2001, 291, 633. 11158672 10.1126/science.291.5504.633

[34] H. Bai , J. Ju , Y. M. Zheng , L. Jiang , Adv. Mater. 2012, 24, 2786. 22737703 10.1002/adma.201200289

[35] J. L. Zhang , Y. C. Han , Langmuir 2007, 23, 6136. 17444664 10.1021/la063376k

[36] H. Bai , X. L. Tian , Y. M. Zheng , J. Ju , Y. Zhao , L. Jiang , Adv. Mater. 2010, 22, 5521. 21104810 10.1002/adma.201003169

[37] Z. W. Yao , M. J. Bowick , Soft Matter 2012, 8, 1142.

